# The Development of Cognitive Reappraisal From Early Childhood Through Adolescence: A Systematic Review and Methodological Recommendations

**DOI:** 10.3389/fpsyg.2022.875964

**Published:** 2022-06-22

**Authors:** Cynthia J. Willner, Jessica D. Hoffmann, Craig S. Bailey, Alexandra P. Harrison, Beatris Garcia, Zi Jia Ng, Christina Cipriano, Marc A. Brackett

**Affiliations:** Yale Center for Emotional Intelligence, Yale Child Study Center, Yale University School of Medicine, New Haven, CT, United States

**Keywords:** cognitive reappraisal, emotion regulation, child development, childhood, adolescence, systematic review

## Abstract

Cognitive reappraisal is an important emotion regulation strategy that shows considerable developmental change in its use and effectiveness. This paper presents a systematic review of the evidence base regarding the development of cognitive reappraisal from early childhood through adolescence and provides methodological recommendations for future research. We searched Scopus, PsycINFO, and ERIC for empirical papers measuring cognitive reappraisal in normative samples of children and youth between the ages of 3 and 18 years published in peer-reviewed journals through August 9th, 2018. We identified 118 studies that met our inclusion criteria. We first present a quantitative review of the methodologies used to investigate cognitive reappraisal in children and adolescents, with attention to variations in methodologies by the sample age range. We then present a qualitative review of findings with attention to: (1) the age at which children begin to effectively use cognitive reappraisal to regulate their emotions, and (2) developmental changes in cognitive reappraisal from early childhood through adolescence. We consider how methodological differences may contribute to inconsistencies in findings, highlight gaps in the literature that remain to be addressed, and make recommendations for future directions.

## Introduction

Emotion regulation refers to “the processes by which individuals influence which emotions they have, when they have them, and how they experience and express these emotions” (Gross, 1998, p. 275). One of the more widely studied forms of emotion regulation—*cognitive reappraisal*—involves changing one's perception of the meaning or self-relevance of a situation to change its emotional impact (Gross, 2015). Most studies of reappraisal measure either the frequency with which people use this strategy in their daily live or the *effectiveness* with which people can use this strategy to modulate their emotions. Reappraisal effectiveness is conceptualized as the extent to which reappraisal attempts are successful at either reducing an unpleasant or unwanted emotion or enhancing a pleasant or wanted emotion.

Extensive research has highlighted cognitive reappraisal as an adaptive emotion regulation strategy (Augustine and Hemenover, 2009; Webb et al., 2012; Gross and Thompson, 2014). Among adults, frequent use of reappraisal is linked to indicators of psychological health and wellbeing, including positive shifts in affect, greater life satisfaction, better interpersonal relationships, and fewer symptoms of psychopathology (Gross and John, 2003; Haga et al., 2009; Aldao et al., 2010).

Despite numerous empirical studies of cognitive reappraisal in child and adolescent samples, findings regarding the normative development of reappraisal have not yet been systematically synthesized. Specifically, at what age does cognitive reappraisal emerge as an effective emotion regulation strategy? How does its use and effectiveness change with development? These questions have important implications for mental health practitioners, educators, and parents who wish to support children's and adolescents' use of developmentally suitable and effective emotion regulation strategies.

### Reappraisal and Cognitive Development

Existing research on cognitive development provides good reasons to expect substantial developmental change in the ability to effectively use cognitive reappraisal to modulate one's emotions. Across childhood, children demonstrate increasing awareness that they can change their interpretation of a situation to modify their emotions (Flavell et al., 2001; Stegge and Terwogt, 2007; Bamford and Lagattuta, 2012). This awareness may be necessary for children to independently and flexibly deploy cognitive reappraisal to manage their emotions. Reappraisal is also a cognitively demanding strategy that is dependent on underlying executive functions such as working memory and attentional set shifting (McRae et al., 2012b). Cognitive and neurophysiological assessments show that executive functions evolve rapidly in early childhood and continue to strengthen significantly throughout childhood and adolescence (Gogtay et al., 2004; Casey et al., 2005; Best and Miller, 2010; Luna et al., 2010). The effectiveness of cognitive reappraisal may show a similar developmental trajectory.

### Measurement of Cognitive Reappraisal

A variety of methodologies have been used to measure cognitive reappraisal in child and adolescent samples. These include self- and parent-report questionnaires, observations of spontaneous self-talk, open-ended interviews, assessments of responses to hypothetical vignettes, and emotion regulation tasks with instructions to reappraise emotional stimuli. Each assessment methodology sheds light on a different facet of reappraisal and has a unique set of psychometric strengths and weaknesses. For example, self-report questionnaires provide insight into respondents' subjective experiences but are limited by respondent biases (Duckworth and Yeager, 2015). Emotion regulation tasks are more objective but have questionable ecological validity. Conclusions about the developmental course of reappraisal are inherently bounded by the limitations of the methodologies used to assess reappraisal. Moreover, some assessment methodologies are more appropriate for use with certain age ranges, which in turn influences the quality and interpretation of the data available on reappraisal in different age groups.

### The Present Study

In this paper, we present a systematic review of the evidence base regarding the development of cognitive reappraisal from early childhood through late adolescence. We first examine the methodologies used to measure cognitive reappraisal in children and adolescents. Given our focus on developmental change, we address how assessment methodologies vary depending on the age range under study. We then provide a narrative review of the findings from the literature with attention to: (1) at what age children begin to effectively use cognitive reappraisal to regulate their emotions, and (2) developmental changes in cognitive reappraisal from early childhood through late adolescence. We discuss how methodological differences may contribute to some apparent inconsistencies in findings, and we highlight gaps in the literature that remain to be addressed.

## Methods

We conducted a systematic search of online article databases to identify empirical studies measuring cognitive reappraisal in normative samples of children and youth between the ages of 3 and 18 years published in peer-reviewed journals through August 9th, 2018. After eligibility screening, we identified 118 studies that met our inclusion criteria. We coded each study for features of the participant sample and methodology, including the age range of participants, type of assessment used, and the emotion that was the target of reappraisal. A table of all coded studies is provided in the Supplementary Materials.

### Literature Search and Article Eligibility Screening

**Figure 1:**
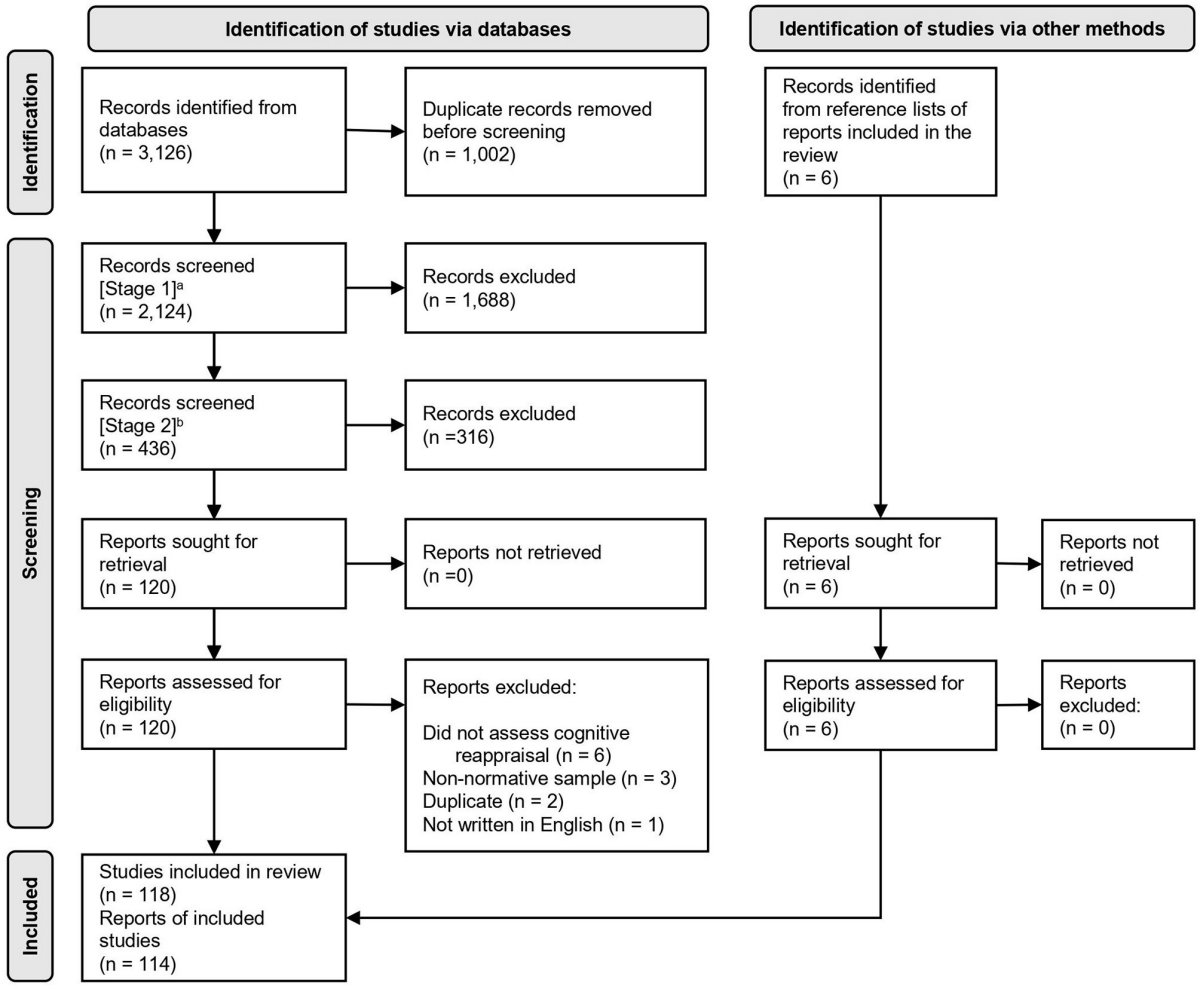
Study identification and screening flow diagram. ^a^In the first screening stage, records were retained if they were an empirical study (not a review) of the knowledge, choice, or use of specific emotion regulation strategies in children and/or adolescents between the ages of 3 and 18 years; they used a normative developmental sample; they were published in a peer-reviewed journal; and they were written in English. ^b^In the second screening stage, articles were retained if the title or abstract contained the words “reappraisal,” “cognitive restructuring,” “reframing,” “re-evaluating,” or “cognitive emotion regulation”.

The studies included in this review were identified using a two-stage process focusing first on identifying empirical studies of children's and adolescents' emotion regulation strategies and then narrowing to focus solely on cognitive reappraisal. Following the Preferred Reporting Items for Systematic Reviews and Meta-Analyses (PRISMA) guidelines (Page et al., 2021), a flow diagram of the study identification and screening process is provided in Figure 1. We searched Scopus, PsycINFO, and ERIC for empirical articles on emotion regulation strategies in children and adolescents. We searched for articles with titles or abstracts that included the term “emotion^*^” and related words (e.g., “affect^*^”) as well as specific emotions (e.g., “anger,” “sadness,” “disappointment”) within two words of the term “regulat^*^” and related terms (e.g., “manag^*^,” “control”). These searches included the required term “strateg^*^” to limit the results to articles discussing emotion regulation strategies. We also searched for specific emotion regulation strategies by name (e.g., “cognitive reappraisal,” “expressive suppression,” “positive self-talk”). In PsycINFO and ERIC, we used database filters to limit the search results to articles with child and adolescent samples; in Scopus, we used search terms (e.g., “child^*^,” “adolesc^*^”, “youth”) to limit the results to these samples. We used database filters to restrict the results to peer-reviewed journal articles published in English. The full search terms and filters used are provided in the Supplementary Materials. A total of 2,124 unique articles were identified by these database searches.

In the first record screening stage, one post-baccalaureate research associate (A.P.H.) manually reviewed the titles and abstracts of all identified records for the following inclusion criteria:

1. Empirical study (not a review) of the knowledge, choice or use of specific emotion regulation strategies in children and/or adolescents between the ages of 3 and 18 years
2. Used a normative developmental sample (i.e., not selected based on a clinical diagnosis, cutoffs on a clinical screener, or exposure to a particular trauma or stressful life experience)
3. Published in a peer-reviewed journal
4. Written in English

In cases where the research associate was unsure whether a record met the inclusion criteria, a determination was made in consultation with the first author. This first screening stage resulted in the retention of 436 records.

In the second screening stage, we narrowed our focus to studies of cognitive reappraisal. Using EndNote's library search feature, we searched the titles and abstracts of records retained from the first screening stage for the terms “reappraisal,” “cognitive restructuring,” “reframing,” “re-evaluating,” and “cognitive emotion regulation.” Records were only retained if their title or abstract included any of these search terms (*n* = 120). We obtained the full-text reports for all retained records. Two post-baccalaureate research associates (A.P.H. and B.G.) then manually reviewed the full-text reports for the following inclusion criteria:

1. Empirical study (not a review) of cognitive reappraisal in children and/or adolescents between the ages of 3 and 18 years
2. Used a normative developmental sample (i.e., not selected based on a clinical diagnosis, cutoffs on a clinical screener, or exposure to a particular trauma or stressful life experience)
3. Published in a peer-reviewed journal
4. Written in english.

In cases where the research associates were unsure whether a study met the inclusion criteria, a determination was made in consultation with the first author. This second screening stage resulted in the retention of 108 reports. Six additional eligible reports were identified because they were cited in other reports included in this review, resulting in a total of 114 reports. Four of these reports each described two independent studies, resulting in 118 studies being included in the systematic review.

### Study Coding

Two post-baccalaureate research associates with undergraduate psychology degrees (A.P.H. and B.G.) independently coded the included studies for the sample age range(s), the type of assessment used to measure reappraisal, and the emotion that was the target of reappraisal. Twenty-eight studies (24%) were coded by both research associates to establish inter-rater reliability. The coders held weekly meetings with the first author to review double-coded studies and discuss any coding questions. Any disagreements or uncertainties in the coding of the studies were resolved by consensus.

#### Sample Age Range

We coded whether the study included participants in each of the following age ranges: early childhood (ages 3–6 years), middle childhood (ages 7–10 years), early adolescence (ages 11–14 years), and late adolescence (ages 15–18 years). The inclusion of an age range was coded based on the minimum and maximum ages of the sample, if reported; otherwise, the sample age ranges were estimated based on the mean age of the sample ±2.50 (*SD*_*age*_). If papers did not report participants' ages but reported schooling level, age ranges were inferred from the schooling level of participants (e.g., high school samples were coded as “late adolescence”), with attention to schooling age norms for the country in which the study was conducted. Studies with samples that spanned multiple age ranges were positively coded in all applicable age ranges.

#### Assessment Type

We coded the types of assessments used to measure reappraisal in each study. Assessments were coded as: questionnaire (self- or parent-report), emotion regulation task with self-reported affect and/or physiological/neural outcome measures, observation, vignette-based assessment, interview, experience sampling self-report, or daily diary. Definitions and examples of each assessment type are provided in Table 1.

**Table 1:** Coding categories for reappraisal assessment type.

**Assessment type**	**Definition**	**Example**
Self-report questionnaire	Closed- or open-response questions asking children/youth to report on their use of reappraisal, in general or in response to a particular kind of situation	Emotion regulation questionnaire (Gross and John, 2003), e.g., “I control my emotions by changing the way I think about the situation I'm in”
Parent-report questionnaire	Closed- or open-response questions asking parents to report on their child's use of reappraisal	Parent-rated emotion regulation questionnaire (Gunzenhauser et al., 2017)
Emotion regulation task with self-reported affect outcome	Direct assessment of the effect of instructions to use reappraisal, or self-reported use of reappraisal, on self-reported affective responses to emotion-eliciting stimuli or an emotion induction	Differences in self-reported negative affect after viewing affective images paired with instructions to reappraise versus “just look at” the image (e.g., McRae et al., 2012b)
Emotion regulation task with physiological/neural outcome	Direct assessment of the effect of instructions to use reappraisal, or self-reported use of reappraisal, on physiological or neural (fMRI or EEG) responses to emotion-eliciting stimuli or an emotion induction	Differences in fMRI activation patterns while viewing affective images paired with instructions to reappraise vs. “just look at” the image (e.g., McRae et al., 2012a)
Observation	Observer coding of participants' behavior and/or audible self-talk in either naturalistic or laboratory contexts	Coding of children's verbalizations during a laboratory-based disappointment task (e.g., Morris et al., 2011)
Vignette-based assessment	Questions about what the participant would do/think, or what someone *should* or *could* do/think, in specific hypothetical scenarios	Coding of children's suggestions for how the protagonists in situational vignettes could make a negative feeling “go away” (Davis et al., 2010)
Interview	Interview about what the participant does/thinks or did/thought, grounded in their lived experiences	Structured interview about youths' strategies for coping with stress (Shaunessy-Dedrick et al., 2015)
Experience sampling self-report	Closed- or open-response questions asking children/youth to report on their use of reappraisal, answered multiple times a day regarding their current or very recent experiences	Five times a day over two consecutive weeks, participants rate their current affect and their use of reappraisal since their previous response (Le Vigouroux et al., 2017)
Daily diary	Daily structured diaries in which youth report on what they experienced/did/thought that day	Daily diary of stressors experienced and coping strategies used over the last 24 h (Valiente et al., 2015)

#### Target Emotion

Where reported, we coded the discrete emotion or emotional state that was the target of reappraisal. We specified an initial set of discrete emotion categories *a priori* (i.e., fear, anger, sadness, happiness, frustration, anxiety, disappointment, boredom, excitement) and incorporated additional categories as they emerged during the article coding process (i.e., desire/craving, disgust, shame, guilt, pain, and social exclusion).

The majority of studies did not focus on discrete emotions or emotional states as the target of reappraisal. We coded these studies as targeting “general negative emotions,” “general positive emotions,” or “nonspecific emotion.” Studies coded as targeting “general negative emotions” assessed reappraisal of negatively-valenced stimuli or negative situations. These included, for example, studies using the Cognitive Emotion Regulation Questionnaire (Garnefski et al., 2002), which asks respondents to report on their cognitions following “negative or unpleasant events,” and emotion regulation tasks in which participants were instructed to reappraise negatively-valenced affective images. Studies coded as targeting “general positive emotions” measured reappraisal of positively-valenced stimuli or situations (only two studies were coded in this category). Finally, studies coded as targeting “nonspecific emotion” assessed the use of reappraisal without differentiating between positively and negatively valanced emotions. Nearly all of the studies coded in this category used the Emotion Regulation Questionnaire (ERQ; Gross and John, 2003) or an adaptation of the ERQ for children and adolescents (ERQ-CA; Gullone and Taffe, 2012), both of which ask about respondents' use of reappraisal to both up-regulate positive emotions and down-regulate negative emotions but do not yield distinct subscales for the regulation of positive vs. negative emotions.

#### Inter-rater Reliability

Twenty-eight studies (24%) were independently coded by both research associates to establish inter-rater reliability. Many coding categories had a low prevalence of positive codes due to the natural distribution of the data. Given that the commonly-used Cohen's Kappa statistic is known to be misleading under these conditions (Byrt et al., 1993; McHugh, 2012) and is inestimable in cases where there is no variance in the coded values (e.g., 100% agreement that a condition is not present), we examined inter-rater reliability for all categorically coded variables using percent agreement. Percent agreement for all categorically coded variables was between 93 and 100%.

### Analytic Approach

We first examined descriptive statistics of the included studies' sample characteristics, the type of assessment used to measure reappraisal, and the target emotion of reappraisal. Given our interest in age-related differences, we explored variations in reappraisal assessment methodologies by the age range(s) included in the sample. Finally, we conducted a qualitative review of the content of the identified studies with a focus on evidence regarding (1) at what age children begin to use reappraisal effectively and (2) how the use and effectiveness of reappraisal as an emotion regulation strategy change from middle childhood through late adolescence. We provide a narrative summary of our findings with attention to how assessment methodologies may influence the conclusions that can be drawn.

## Results

### Characteristics of Included Studies

A total of 118 studies met the eligibility criteria for inclusion in this review, over 90% of which were published in the year 2010 or later (Table 2).

**Table 2:** Characteristics of studies included in the systematic review.

**Characteristic**	***n*** **(%)**
**Year of publication**	
Before 2000	2 (2%)
2000–2009	9 (8%)
2010–2018	107 (91%)
**Sample age range(s)**	
Early childhood (3–6.9 years)	22 (19%)
Middle childhood (7–10.9 years)	45 (38%)
Early adolescence (11–14.9 years)	88 (75%)
Late adolescence (15–18.9 years)	78 (66%)
**Reappraisal assessment type(s)**	
Self-report questionnaire	76 (64%)
Emotion regulation task	32 (27%)
Self-reported affect	26 (22%)
Physiological/neural measures	20 (17%)
Observation	3 (3%)
Vignette	4 (3%)
Interview	3 (3%)
Parent-report questionnaire	3 (3%)
Experience sampling self-report	2 (2%)
Daily diary	1 (1%)
**Emotion(s) targeted**	
Nonspecific emotion	42 (36%)
General negative emotions	55 (47%)
General positive emotions	2 (2%)
Discrete negative emotions^a^	21 (18%)
Desire/craving	3 (3%)
Total *N*	118

*Sample age range(s), reappraisal assessment type(s), and emotion(s) targeted were coded non-exclusively. Therefore, percentages for these categories do not sum to 100*.

a *Discrete negative emotions included sadness (n = 13), anger (n = 10), fear (n = 7), disgust (n = 2), shame (n = 2), anxiety (n = 1), disappointment (n = 1), frustration (n = 1), guilt (n = 1), pain (n = 1), and social exclusion (n = 1)*.

#### Study Sample

The average study sample size was 506 participants (median = 177, range = 14–4,316). A greater number of study samples included youth in early and late adolescence (*n* = 88 and 78, respectively) relative to middle childhood (*n* = 45) and early childhood (*n* = 22; Table 2).

About 35% of studies were conducted in North America (The United States, *n* = 36, and Canada, *n* = 4). Another 41 studies (35%) were conducted in Europe, most notably The Netherlands (*n* =10), Italy (*n* = 6), Germany (*n* = 5), England (*n* = 5), and Belgium (*n* = 4). Sixteen percent of studies were conducted in East Asia (China, *n* = 14, Taiwan, *n* = 3, and Hong Kong, *n* = 1), and 12% of studies were conducted in Australia or New Zealand (*n* = 13 and 1, respectively). A single study was conducted in each of Argentina, Brazil, Israel, Turkey, and Pakistan. Thus, while children's and adolescents' cognitive reappraisal has been studied widely internationally, wealthy and highly industrialized countries are overrepresented.

The majority of studies (*n* = 84; 71%) did not report on the racial and/or ethnic identities of their participants, or only reported the racial/ethnic identity of a small subsample. The proportion of studies reporting participants' racial/ethnic identities varied widely by country (e.g., 56% of studies in the United States vs. 29% of studies in China). For the 34 studies that reported their sample's racial/ethnic composition (mostly from the United States [*n* = 20], China [*n* = 4], England [*n* = 3], and Canada [*n* = 3]), data is reported in Supplementary Table 1. A review of reported participant racial and/or ethnic identities indicates that more work is required to ensure the evidence base on cognitive reappraisal in childhood and adolescence is representative of diverse racial and ethnic identities. For example, participants identifying as Black or of African heritage only constituted more than 20% of the sample in nine studies and in no case did they constitute more than 48% of the sample. Furthermore, participants identifying as Latino or of Hispanic, South or Central American heritage only constituted more than 20% of the sample in two studies, and in no case did they constitute more than 54% of the sample.

#### Assessment Type

Self-report questionnaires were the most frequently used methodology for assessing reappraisal (*n* = 76; 64% of studies; Table 2). Self-report questionnaires were particularly predominant among studies with adolescent samples (used by nearly 80% of these studies; Table 3), whereas they were used by only 42% of studies with middle-childhood samples, and none of the studies with early childhood samples. This pattern presumably reflects both the greater age-appropriateness of self-report questionnaires for adolescents who have greater metacognitive awareness and the relative ease of using self-report questionnaires in large samples of youth relative to more costly and burdensome assessments such as laboratory-based emotion regulation tasks.

**Table 3:** Types of assessments used to measure reappraisal, by age ranges included in the study sample.

**Assessment type**	**Age range**				***n*** **(%)**
	**Early childhood**	**Middle childhood**	**Early adolescence**	**Late adolescence**	
Self-report questionnaire	0 (0%)	19 (42%)	67 (76%)	61 (78%)	76 (64%)
Emotion regulation task	13 (59%)	24 (53%)	20 (23%)	15 (19%)	32 (27%)
Self-report affect	8 (36%)	19 (42%)	20 (23%)	15 (19%)	26 (22%)
Physiological/neural measures	8 (36%)	14 (31%)	10 (11%)	10 (13%)	20 (17%)
Observation	2 (9%)	2 (4%)	1 (1%)	0 (0%)	3 (3%)
Vignette	3 (14%)	1 (2%)	1 (1%)	1 (1%)	4 (3%)
Interview	2 (9%)	1 (2%)	1 (1%)	1 (1%)	3 (3%)
Parent-report questionnaire	3 (14%)	2 (4%)	0 (0%)	0 (0%)	3 (3%)
Experience-sampling self-report	0 (0%)	0 (0%)	2 (2%)	2 (3%)	2 (2%)
Daily diary	0 (0%)	1 (2%)	1 (1%)	1 (1%)	1 (1%)
*N*	22	45	88	78	118

*Percentages are calculated with the total number of studies including participants in each age range as the denominator. Studies with samples that spanned multiple age ranges are coded positively for each relevant age range, and studies that used multiple assessment types are coded positively for each assessment type used. Therefore, percentages do not sum to 100%. Early Childhood = 3–6.9 years old, Middle Childhood = 7–10.9 years old, Early Adolescence = 11–14.9 years old, Late Adolescence = 15–18.9 years old*.

Most studies that administered self-report questionnaires used the Emotion Regulation Questionnaire (ERQ; Gross and John, 2003) or the Cognitive Emotion Regulation Questionnaire (CERQ; Garnefski et al., 2001) [ERQ: 55%; CERQ: 23%]. The reappraisal subscale of the ERQ consists of 6 items asking whether respondents typically control their emotions by changing their thoughts. The CERQ includes two cognitive reappraisal subscales: *positive reappraisal*, consisting of four items focused on finding a positive side to a negative situation, and *putting into perspective*, consisting of four items focused on thinking that the situation could have been worse.

Emotion regulation tasks were the second most frequently used assessment methodology (*n* = 32; 27% of studies; Table 2), used by 59% of studies with early childhood samples, 53% of studies with middle-childhood samples, and roughly 20% of studies with early or late adolescent samples (Table 3). The lower proportion of studies with adolescent samples using this methodology is presumably due, again, to the greater ease of using self-report questionnaires.

Among studies using emotion regulation tasks, most used a paradigm in which participants viewed emotion-eliciting stimuli (e.g., images or video clips) paired with instructions to either cognitively reappraise the stimulus or to passively view and react naturally. The effectiveness of cognitive reappraisal was operationalized as the difference in self-reported affect and/or neural measures between the reappraisal and comparison conditions. Of the studies that used neural measures, many focused on functional magnetic resonance imaging (fMRI) of activation in the amygdala, a region of the brain that assigns affective significance to stimuli and orchestrates emotional responses in the brain and body (LeDoux and Phelps, 2008). Other studies utilized electroencephalography (EEG) to measure the amplitude of the Late Positive Potential (LPP), a slow positive voltage change in the event-related potential that correlates with the affective intensity of stimuli (Cuthbert et al., 2000). Reductions in amygdala activation or in LPP amplitudes are generally interpreted as indicating successful down-regulation of emotional arousal.

Only five studies used both an emotion regulation task *and* a self-report questionnaire, suggesting that more research is warranted to cross-validate these commonly used assessment methodologies. Finally, only a handful of studies used observational (*n* = 3), vignette-based (*n* = 4), open-ended interview (*n* = 3), parent-report questionnaire (*n* = 3), experience sampling self-report (*n* = 2), or daily diary (*n* = 1) methods to assess reappraisal in children or adolescents (Table 2). More research is warranted to explore the utility of these methods for studying cognitive reappraisal.

#### Target Emotion

Studies most frequently assessed reappraisal targeting general negative emotions (*n* = 55; 47% of studies) or nonspecific emotions (*n* = 42; 36% of studies; Table 2). A smaller number of studies (*n* = 21; 18%) assessed reappraisal targeting discrete negative emotions such as sadness or anger. Three studies assessed reappraisal targeting desire or craving, and two studies assessed reappraisal targeting general positive emotions. Only four studies separately examined reappraisal targeting two or more discrete emotions within the same study. This suggests there is more work to be done to explore how children's and adolescents' use of reappraisal may differ depending on the kind of emotion being reappraised.

### Findings on the Emergence of Cognitive Reappraisal

In this section, we provide a narrative summary of findings from the studies included in our systematic review regarding the age at which children begin to be able to effectively use cognitive reappraisal to regulate their emotions. Reflecting the methodologies used to assess reappraisal in early childhood (Table 3), this section focuses on studies using emotion regulation tasks, observations, interviews, and vignette-based assessments.

**Observational and vignette-based interview studies suggest that children begin to use reappraisal as an emotion regulation strategy between the ages of 3 and 5 years with adult scaffolding**. In an observational study of preschool-aged children, Stansbury and Sigman (2000) found that roughly 65% of 3-year-old and 85% of 4-year-old children expressed at least one verbalization or behavioral response in frustration induction tasks that indicated use of reappraisal (e.g., turning clean-up into a game), though most of these instances were scaffolded by their parents. In an observational study of children's responses to a laboratory-based disappointment task, Morris et al. (2011) observed that joint mother-child cognitive reappraisal resulted in reduced expressions of anger and sadness in children as young as 4 years old. In contrast, Sala et al. (2014) observed that, in a sample of children aged 3–6 years, children aged 3–4 years did not generate any cognitive reappraisals in a story completion task, and the number of reappraisals generated increased with age among children aged 5–6 years. In a vignette-based interview study by Davis et al. (2010), roughly 69% of children aged 5–6 years offered metacognitive emotion regulation strategies, including some instances of positive reappraisal.

In sum, it appears that across observational and vignette-based interview tasks, some preschoolers as young as 3 years old can engage in cognitive reappraisal with adult guidance, and by age five some children may independently generate cognitive reappraisals. Moreover, some observational evidence suggests that parent-scaffolded reappraisal can be effective at modulating young children's emotional expression.

**Studies measuring self-reported affect during emotion regulation tasks show that by around 7 or 8 years of age—and possibly as young as 6—children report reduced negative affect when they are instructed to reappraise negative stimuli**. Studies using directed reappraisal paradigms, in which children are provided with a story for interpreting each stimulus, show that children around 7 or 8 years old report lower negative affect following negative stimuli that are paired with a reappraisal story (Pitskel et al., 2011; Dougherty et al., 2015; Leventon and Bauer, 2016; Van Cauwenberge et al., 2017). Furthermore, studies using non-directed reappraisal paradigms have revealed that, by middle childhood, children report reduced negative affect when they generate and use their own reappraisals for emotional stimuli. For example, reductions in self-reported negative affect following reappraisal of sad stimuli have been found in samples of children aged 8–10 years (Lévesque et al., 2004) and 8–12 years (Belden et al., 2014). There is also substantial evidence that, by the age of 10 years, children reliably report reduced negative affect when instructed to reappraise negative stimuli (Silvers et al., 2012, 2015, 2017; Schienle et al., 2015).

Only a few studies have used emotion regulation tasks with self-reported affect measures with children as young as 6 years, and we did not locate any studies using self-reported affect with children younger than 6 years. In a sample of 105 individuals aged 6–23 years, Silvers et al. (2014) found that children as young as 6 years old who were instructed to use psychological distancing (a form of reappraisal) reported reduced food craving to appetitive food stimuli. In contrast, Silvers et al. (2017) found in a separate sample of 6–23 year-olds that children under the age of 10 were ineffective in using psychological distancing to reduce their self-reported negative affect to negative social stimuli. In a sample of 126 children aged 6–13 years, Davis (2016) observed that, after viewing a sad film clip, children who were instructed to reappraise the content of the clip reported greater reductions in sadness relative to children who were instructed to ruminate on the content, and no significant age differences in the experimental condition effects were observed. However, in this study, children in a control condition who received no instructions on how to think about the film clip showed similar reductions in sadness as did those who were instructed to use reappraisal.

The above findings must be interpreted in light of important limitations in the validity of affective self-report in children. Such self-reports rely on participants' introspective awareness of their emotions. This is particularly problematic for developmental research since younger children may have less insight into their emotional experiences than do older children and adolescents. In support of this point, Van Cauwenberge et al. (2017) observed that the younger children in their sample (those aged 8–11 years) showed a greater effect of reappraisal on self-reported negative affect despite showing lesser effects of reappraisal on *neural* indices of emotional reactivity relative to adolescents aged 12–15 years. Additionally, since the instruction to reappraise is given explicitly, participants' self-reports are subject to social desirability biases—that is, they may report reduced negative affect in reappraisal conditions because they know that is the desired response. These limitations inherent in subjective self-reports of affective states may be overcome by more objective neural and psychophysiological measures of emotional reactivity.

**Contrary to findings from emotion regulation tasks using affective self-report, studies using emotion regulation tasks with *neural and psychophysiological measures* provide mixed evidence of reappraisal effectiveness in early and middle childhood**. In adults, there is robust evidence that reappraisal of negative images decreases both amygdala activation (Buhle et al., 2013) and LPP amplitudes (Hajcak and Nieuwenhuis, 2006). In children, however, the evidence is less robust. Some studies have observed significant LPP reductions—indicating reduced emotional arousal—during reappraisal of negative images in samples of children aged 4–5 years (Hua et al., 2015), 7–10 years (Dennis and Hajcak, 2009), and 8 years (Leventon and Bauer, 2016), as well as decreased amygdala activation during reappraisal of sad images in children aged 8 to 12 years (Belden et al., 2014). In the Leventon and Bauer (2016) study, the effect of reappraisal on the LPP was even maintained when the same images were viewed again several days later. Additionally, Davis et al. (2016) observed that children aged 5–6 years who were instructed to use reappraisal to manage their emotions while watching sad and scary film clips demonstrated greater respiratory sinus arrhythmia augmentation compared to those in the control condition, suggesting enhanced parasympathetic regulation of negative emotions.

In contrast, several studies have shown no significant effect of reappraisal on LPP amplitudes to negative images in samples of children aged 5–7 years (DeCicco et al., 2012), 7–9 years (DeCicco et al., 2014), and 8–11 years (Van Cauwenberge et al., 2017), and no reduction in amygdala activation with reappraisal of sad film clips in children aged 8–10 years (Lévesque et al., 2004). Contrary to expectation, some studies have even provided evidence of *increased* emotionality during reappraisal of negative images in children, including enhanced amygdala activation in children aged 6–9 years (Dougherty et al., 2015; Silvers et al., 2017) and enhanced LPP amplitudes in children aged 8–9 years (Van Cauwenberge et al., 2017).

The reason for the inconsistencies across these studies is not clear, but individual differences in the reappraisal abilities of children likely play a role. Notably, although DeCicco et al. (2012) found no effect of reappraisal on the LPP in a sample of 5–7 year-old children, Babkirk et al. (2015) found in the same sample considerable variability in children's LPP responses: some children exhibited increased LPP amplitudes during reappraisal suggesting heightened emotionality, whereas other children exhibited decreased LPP amplitudes during reappraisal suggesting reduced emotionality. Decreased LPP amplitudes during reappraisal were in turn associated with adaptive emotion regulation strategy use during waiting and disappointing tasks 2 years later. Additionally, DeCicco et al. (2014) found in their sample of children aged 7–9 years that the magnitude of the LPP reduction by reappraisal increased with greater age in months, suggesting that there may be an inflection point around 8 years of age when children begin to be able to use directed reappraisals to effectively modulate their emotional responses to negative images. It has also been proposed that the typical reappraisal task may place too heavy a demand on working memory for some young children (Hua et al., 2015). Thus, it is possible that inconsistencies across studies could be explained by differences in the developmental maturity, executive function, or emotion regulation skills of the children included in the sample, or by differences in experimental design details. Moreover, many studies using emotion regulation tasks with children in this age range use samples of about 15 to 30 children (Lévesque et al., 2004; Dennis and Hajcak, 2009; DeCicco et al., 2012, 2014; Babkirk et al., 2015; Dougherty et al., 2015). Studies with larger samples would yield more reliable evidence regarding the influence of reappraisal on neural indices of emotionality in children.

**Studies using emotion regulation tasks with neural and psychophysiological measures provide somewhat more consistent evidence of reappraisal effectiveness by mid to late adolescence**. Van Cauwenberge et al. (2017) observed that significant LPP reductions with reappraisal emerged in children aged 12–15 years, whereas reappraisal-related LPP modulations were not significant in children aged 8–11 years. Similarly, Silvers et al. (2015) found that reappraisal significantly down-regulated amygdala activation in a subgroup of adolescents aged 14–17 years, whereas it did not significantly modulate amygdala activation in a subgroup of children aged 10–13 years. A couple studies that examined trends in reappraisal effectiveness across adolescence and into young adulthood found that significant amygdala down-regulation with reappraisal emerged in late adolescence (Stephanou et al., 2016; Silvers et al., 2017). In a sample of adolescents with a mean age of 14.7 years (SD = 0.80), Vögele et al. (2010) observed lower cardiac reactivity to anger provocation among individuals who spontaneously engaged in reappraisal vs. those who engaged in anger rumination. In contrast, one study in a sample of adolescents and young adults aged 10–22 years found no significant effect of reappraisal on amygdala activation across the entire sample and no age differences in this effect (McRae et al., 2012a). A separate study with adolescents aged 12–15 years found that reappraisal instructions did not alter fear-potentiated startle reflexes (Shore et al., 2017), and another small study of 21 adolescents found no differences in activation in reward-related brain regions with instructions to reappraise appetitive food stimuli (Yokum and Stice, 2013).

### Developmental Change in Cognitive Reappraisal From Middle Childhood Through Adolescence

In this section, we provide a narrative summary of findings from the studies included in our systematic review regarding developmental changes in the use and effectiveness of cognitive reappraisal from middle childhood through adolescence. Given that the vast majority of studies with children over the age of 7 years used either a self-report questionnaire or emotion regulation task to measure reappraisal (Table 3), we focus on findings from studies using these two methodologies.

**When using self-report survey methodology, the bulk of evidence suggests that the frequency of reappraisal use increases from middle childhood through adolescence and into adulthood**. In a longitudinal daily diary study which assessed children's responses to life stressors every 2 years from roughly ages 9–15 years, children's use of positive reappraisal was found to increase with age (Valiente et al., 2015). Similarly, Tu et al. (2016) found that older children reported greater use of cognitive reappraisal and reframing strategies in response to their parents' marital conflict than did younger children. Garnefski and Kraaij (2006) found that positive reappraisal was reportedly used less by younger adolescents than by older adolescents and less by older adolescents than by adults. Similarly, Wilson and Hall (2012) observed lower self-reported use of positive reappraisal by adolescents vs. adults and, using an experience-sampling self-report methodology, Le Vigouroux et al. (2017) observed that use of positive reappraisal increased with age from 13 to 80 years old.

In contrast, a couple of studies have found decreases in self-reported use of reappraisal with age across adolescence. Gullone et al. (2010) collected self-reports of reappraisal use in 1,128 children and adolescents ages 9–15 years, including two follow-up assessments each 1 year apart. They observed less frequent use of reappraisal in older compared to younger participants at the first assessment occasion, although there was no significant change in reappraisal frequency within individuals across the 2-year follow-up period. Boyes et al. (2016) also found a very small but significant negative correlation between age and self-reported use of cognitive reappraisal in a sample of over 2,637 youth between the ages of 12 and 18 years. The reason for these inconsistent findings is not clear. Given the large sample sizes of these contradictory studies, further research is warranted to confirm whether self-reported reappraisal frequency increases or decreases from middle childhood through adolescence.

**Studies measuring self-reported affect during emotion regulation tasks provide mixed evidence on differences in reappraisal *effectiveness* from middle childhood through adolescence**. Whereas, self-report questionnaires yield data on respondents' perceived frequency of using reappraisal, emotion regulation tasks assess participants' ability to effectively use reappraisal to modulate their affective responding to emotionally provocative stimuli. In two independent samples of individuals aged 10–23 years, Silvers et al. (2012) observed that the magnitude of reappraisal-related reductions in self-reported negative affect increased linearly between the ages of 10 and 16 or 17 years. In two subsequent independent studies, Silvers and colleagues found that reappraisal-related reductions in self-reported negative affect increased linearly from age 10 through 23 years (Silvers et al., 2015) and from age 6 through 23 years (Silvers et al., 2017).

Other studies have found no significant age differences in the magnitude of reappraisal-related reductions in self-reported negative affect from ages 7 to 17 years (Pitskel et al., 2011), 6 to 13 years (Davis, 2016), 12 to 15 years (Shore et al., 2017), or 15 to 25 years (Stephanou et al., 2016), or in self-reported food craving in response to appetitive food stimuli in a sample of youth aged 6–23 years (Silvers et al., 2014). A couple of studies have even observed *smaller* reappraisal-related reductions in self-reported negative affect in adolescence vs. middle to late childhood. McRae et al. (2012a) found that reappraisal-related reductions in self-reported negative affect declined from ages 10 to 17 years before increasing dramatically in young adulthood, and Van Cauwenberge et al. (2017) observed that 12- to 15-year-old adolescents reported smaller reappraisal-related reductions in negative affect than did 8–11 year old children. As discussed previously, it is possible that younger participants are more susceptible to social desirability biases and therefore may over-report their reduction in negative affect following reappraisal. Subtle differences in the instructions provided to participants across studies—e.g., whether they are explicitly told to use reappraisal to “feel less bad”—may modulate the magnitude of the social desirability bias in different age ranges.

**Contrary to the mixed findings from studies measuring self-reported affect during emotion regulation tasks, the bulk of evidence from studies assessing *neural measures* during emotion regulation tasks suggests that reappraisal effectiveness improves linearly from middle childhood through adolescence**. Neuroimaging studies have documented linear increases in amygdala down-regulation by reappraisal from middle childhood into late adolescence and early adulthood, suggesting that greater age is associated with more effective use of reappraisal to down-regulate amygdala-mediated affective arousal (Pitskel et al., 2011; Silvers et al., 2015, 2017; Stephanou et al., 2016). Linear increases in reappraisal-related amygdala down-regulation with age were observed in samples aged 6–23 years (Silvers et al., 2017), 10–23 years (Silvers et al., 2015), 15–25 years (Stephanou et al., 2016), and 7–17 years (Pitskel et al., 2011). Parallel findings have been observed using EEG; Van Cauwenberge et al. (2017) found that the magnitude of the reappraisal-related LPP amplitude reduction increased linearly with age from 8 to 15 years. We only identified one neuroimaging study that failed to find a positive association between age in middle childhood through adolescence and the magnitude of amygdala down-regulation during reappraisal (McRae et al., 2012a). Notably, in several studies (Pitskel et al., 2011; Stephanou et al., 2016; Van Cauwenberge et al., 2017) the association of age with decreased amygdala activation or LPP amplitude during reappraisal was observed despite a lack of age differences in self-reported affect during reappraisal.

Intriguingly, Silvers et al. (2015) observed that the association of age with reduced amygdala activation during reappraisal was even greater during re-presentation of images that had been reappraised about 30 min previously. This suggests that, across the adolescent years and into young adulthood, reappraisal may have a longer-lasting impact on emotional reactivity to repeated stressors in addition to becoming a more effective strategy for reducing negative emotions in the moment. Further research is warranted exploring age differences in the temporal dynamics of reappraisal.

**Neuroimaging studies provide evidence that increasing reappraisal effectiveness from middle childhood through adolescence is mediated by maturational changes in neural networks supporting cognitive emotion regulation**. Neuroimaging findings provide some evidence regarding mechanisms of an age-related increase in reappraisal effectiveness. Studies have observed that, across adolescence and into early adulthood, there are age-related changes in the recruitment of prefrontal “cognitive control” regions and in functional connectivity between the amygdala and ventral prefrontal regions during reappraisal (Pitskel et al., 2011; McRae et al., 2012a; Silvers et al., 2015, 2017). In one study among individuals aged 10–22 years, age-related increases in reappraisal effectiveness (based on self-reported affect) were accompanied by a linear association of age with greater activation in the left ventrolateral prefrontal cortex (vlPFC) during reappraisal (McRae et al., 2012a). The left vlPFC is involved in various cognitive control functions including response inhibition and the selection of information from memory (Durston et al., 2002; Badre and Wagner, 2007), and is implicated in reappraisal in adult populations (Ochsner and Gross, 2008). This region may play a role in inhibiting automatic appraisals and selecting alternate appraisals, with greater activation with age indicating that older adolescents and young adults more strongly engage these cognitive control processes during reappraisal.

Similarly, in a sample of individuals aged 6–22 years, Silvers et al. (2017) observed that the negative association of age with amygdala activation during reappraisal was mediated by greater activation of the vlPFC. Furthermore, vlPFC activation was more strongly associated with amygdala down-regulation during reappraisal among individuals with more negative functional connectivity between the amygdala and ventromedial prefrontal cortex (vmPFC) during reappraisal, and age was associated with increasingly negative vmPFC-amygdala functional connectivity during reappraisal. This finding is in line with prior evidence of a maturational shift from positive to negative vmPFC-amygdala functional connectivity during emotion regulation between childhood and early adolescence that is hypothesized to play an important role in age-related increases in the inhibitory control of emotions (Gee et al., 2013).

In a sample of individuals aged 10–23 years who were asked to use psychological distancing to reappraise aversive images, Silvers et al. (2015) found that the association of age with greater reduction in amygdala activation during reappraisal was mediated by greater negative functional connectivity between the rostrolateral prefrontal cortex (rlPFC; a region involved in abstract thinking; Dumontheil, 2014) and the amygdala. It is possible that connectivity with the rlPFC was implicated in this study due to the specific cognitive processes involved in using distancing vs. reinterpretation as a reappraisal tactic.

In an exploratory analysis of developmental differences within a small sample of 15 individuals aged 7–17 years, Pitskel et al. (2011) observed *decreased* reappraisal-related activation in various prefrontal regions, including the right medial orbitofrontal cortex, medial PFC, and left inferior frontal gyrus (a region including the vlPFC), with increasing age. The authors interpret this finding as reflecting less effort required to engage in reappraisal with increasing age. Although this finding appears contradictory to findings from other studies of increased activation in specific prefrontal regions, particularly the vlPFC, it is possible that reappraisal-related activation becomes more focal within the vlPFC while the number of different prefrontal regions activated decreases with age as reappraisal efficiency increases.

We only identified one study that failed to find an association of age with activation in the prefrontal cortex or with PFC-amygdala functional connectivity during reappraisal. In a sample of adolescents and young adults aged 15–25 years (*n* = 78), Stephanou et al. (2016) instead found that age-related reductions in activation in the fusiform gyrus during reappraisal mediated the association of age with greater amygdala down-regulation; similarly, fusiform-amygdala functional connectivity during reappraisal decreased with age. Their experimental paradigm departed from others by using purely social stimuli. Given the role of the fusiform face area in modulating amygdala activation to social stimuli, specifically faces (Fairhall and Ishai, 2007; Pujol et al., 2009), they interpreted this finding as suggesting that the heightened salience of social stimuli for younger adolescents may interfere with their reappraisal efforts. Additionally, the failure to identify age-associated differences in PFC activation during reappraisal could be due to the narrower age range of the sample, which excluded children and early adolescents.

Overall, the bulk of evidence suggests that improvements in reappraisal effectiveness from middle childhood through adolescence result from the maturation of amygdala-prefrontal neural networks supporting cognitive emotion regulation, although the specific prefrontal regions implicated in developmental changes in reappraisal may vary based on differences in methodology or sample. Further research should explore the extent to which these findings are dependent on the nature of the stimuli being reappraised (e.g., social vs. non-social content), the emotion being reappraised (e.g., appetitive vs. aversive emotions), the reappraisal tactic used (e.g., distancing vs. reinterpretation; Ochsner and Gross, 2008), and idiosyncratic features of the sample (e.g., frequency of using reappraisal or reappraisal ability).

## Discussion

This systematic review of the literature on cognitive reappraisal in children and adolescents focused on two research questions: (1) at what age do children begin to effectively use cognitive reappraisal to regulate their emotions, and (2) what developmental changes occur in cognitive reappraisal from middle childhood through adolescence? We discuss the main conclusions from our review, consider how assessment methodologies shape and limit our knowledge, highlight gaps in the literature that remain to be addressed, and make recommendations for future studies.

### The Emergence of Cognitive Reappraisal

The answer to our first research question—at what age children begin to be able to effectively use cognitive reappraisal to regulate their emotions—depends on how reappraisal effectiveness is measured. Observational and vignette-based interview studies suggest that children as young as 3 or 4 years may utilize simple reappraisals in emotionally challenging situations with adult scaffolding, and by around 5 or 6 years of age, children can independently generate reappraisals as an emotion regulation strategy. However, it remains unclear whether most young children can effectively utilize cognitive reappraisal to modulate their emotional arousal, particularly without adult scaffolding. Studies assessing self-reported negative affect during laboratory-based emotion regulation tasks find that children as young as 7 or 8 years old report lower negative affect when they are instructed to reappraise negative stimuli. However, these studies have not been conducted with children under 6 years old due to limitations in the validity of young children's affective self-reports. In contrast, studies assessing neural and psychophysiological measures during laboratory-based emotion regulation tasks provide mixed evidence on whether children can effectively use reappraisal to modulate their emotional arousal prior to mid to late adolescence. A quantitative meta-analysis of these studies would be a logical next step to yield clearer conclusions about children's ability to effectively use cognitive reappraisal in laboratory-based emotion regulation tasks.

Uncertainty regarding the validity of children's affective self-reports and the meaning of neural measures complicates the interpretation of discrepant findings across these indices. For example, changes in younger children's affective self-reports when they are instructed to use reappraisal may be more strongly influenced by social desirability biases relative to those of older children (Crandall et al., 1965), thereby artificially inflating the apparent effectiveness of reappraisal in younger children when assessed using self-reported affect. Future studies may explicitly examine and control for this source of bias by asking children to complete a social desirability scale, which measures the tendency to provide socially desirable—but not fully truthful—responses. Additionally, it has been proposed that increased amygdala activation during reappraisal in younger children could reflect arousal due to greater effort required to engage in reappraisal, rather than reduced effectiveness of reappraisal at down-regulating negative affect (Silvers et al., 2017). Such questions regarding the interpretation of neural measures in children undermine confidence in the conclusions drawn from studies using these measures. Studies using a multi-method approach to measuring reappraisal effectiveness—for example, including affective self-report, neural, peripheral psychophysiological, and observational measures—may help to clarify inconsistent findings across these methodologies.

The mixed findings for neural and psychophysiological indices of reappraisal effectiveness in childhood may be due in part to a high level of between-individual variability in children's reappraisal skills. Further investigation of factors that may underly this variability, such as executive functioning, emotional reactivity, and adult modeling of reappraisal, is an important direction for future research. It is likely that cognitive reappraisal emerges as an effective emotion regulation strategy at substantially younger ages for children who have strong executive functioning skills, less intense emotional reactions, and frequent parental modeling of cognitive reappraisal.

### Developmental Change in Cognitive Reappraisal From Middle Childhood Through Adolescence

Our **second** aim was to explore developmental changes in the use and effectiveness of cognitive reappraisal from middle childhood through adolescence. Since the vast majority of studies with samples in these age ranges measured reappraisal with either self-report questionnaires or emotion regulation tasks, our review focused on knowledge gained from these assessment methodologies. With some notable exceptions, most studies using self-report questionnaires have revealed an increase in the reported frequency of using cognitive reappraisal from middle childhood through late adolescence. This trend aligns with other reviews that have noted increases in the use of more sophisticated cognitive emotion regulation strategies, including reappraisal, from middle childhood through adolescence (Riediger and Klipker, 2014). However, contradictory findings were obtained from two large-sample studies using self-report questionnaires with youth aged 9–15 years (Gullone et al., 2010) and 12–18 years (Boyes et al., 2016), suggesting that further research is warranted.

The bulk of evidence from neuroimaging studies using emotion regulation tasks reveals that the effectiveness of reappraisal at down-regulating amygdala activation to negative emotional stimuli increases linearly from middle childhood through adolescence. Combined with findings of increasing frequency of using reappraisal based on self-report questionnaires, this suggests that youth may both become more adept at using reappraisal to manage their emotions and choose to use it more frequently as they progress through middle childhood and adolescence. Longitudinal studies assessing both constructs across childhood and adolescence could more directly test the extent to which reappraisal effectiveness and frequency of use develop in tandem.

In contrast, findings for age-related changes from middle childhood through adolescence in the impact of reappraisal on *self-reported affect* during emotion regulation tasks were mixed, and several studies observed age-related differences in neural indices despite a lack of age-related differences in self-reported affect during reappraisal. This further highlights the issue of differential validity of self-reported affect vs. neural measures for assessing developmental change in reappraisal effectiveness, and the need to clarify the appropriate interpretation of each of these measures in early childhood through adolescence.

Finally, neuroimaging studies have provided evidence suggesting that age-related increases in reappraisal effectiveness from middle childhood through late adolescence are mediated by the maturation of neural networks connecting the amygdala with prefrontal brain regions supporting the cognitive control of emotions. This supports the hypothesis that developmental improvements in reappraisal ability are supported by the maturation of executive functioning skills, which are mediated by many of the same prefrontal brain regions implicated in reappraisal.

### Methodological Limitations of the Evidence Base and Recommendations for Future Research

The vast majority of studies on cognitive reappraisal in children and adolescents have measured either reappraisal frequency using **s**elf-report questionnaires or reappraisal effectiveness using emotion regulation tasks. Both methodologies have important limitations. Subjective response biases are problematic for both self-report questionnaires and for emotion regulation tasks using self-reported affect to measure reappraisal effectiveness. For example, self-reports are subject to social desirability biases, which have been shown to lessen with age across childhood and adolescence (Crandall et al., 1965). Self-reports also rely on the respondent's introspective awareness of their emotional processes, which is lower in younger children (Stegge et al., 2004; Stegge and Terwogt, 2007). Thus, developmental changes in the accuracy of self-reports undermines any conclusions that might be drawn regarding age differences in reappraisal use or effectiveness using self-report methodology. Future studies are warranted explicitly examining the strength of these biases and their contributions to apparent changes in reappraisal use and effectiveness with age.

Emotion regulation tasks that use neural indices of reappraisal effectiveness offer more objective data. However, as discussed above, the interpretation of neural measures is not always straightforward. Furthermore, emotion regulation tasks completed while fMRI or EEG data are being recorded have more limited ecological validity due to the contrived nature of the experimental paradigm (i.e., being explicitly directed to reappraise images or videos) and the unusual context in which the data are collected (i.e., lying as still as possible in an MRI scanner, or sitting as still as possible in a dim room with EEG electrodes affixed to one's scalp). A handful of studies included in this review (e.g., Hodgins and Lander, 1997; Vögele et al., 2010; Rood et al., 2012; Shore et al., 2017; Dorman Ilan et al., 2018) used experimental paradigms with greater ecological validity, such as recalling a stressful experience or undergoing routine venipuncture at a doctor's office. Further research is warranted using emotion regulation paradigms with emotionally evocative situations that are more directly relevant to participants' lives. For example, neural and psychophysiological measures could be collected while children are instructed to reappraise challenging academic problems or disappointing performance feedback. Investigators collecting neural and psychophysiological data should also more consistently address the potential psychological effects of the data collection context on participants' responses, for example by conducting mock data collection prior to the experiment to help young children feel more at ease (de Bie et al., 2010) and collecting data on age differences in the intensity of anxiety induced by the data collection context.

Additionally, more work is needed to disentangle the effects that different experimental design decisions may have on findings from emotion regulation tasks. Experimental details that vary widely across studies include whether participants are provided with a reappraisal story for each stimulus or asked to generate their own reappraisals, the amount of training on reappraisal provided to participants, the reappraisal tactics they are encouraged to use (e.g., positive reappraisal vs. psychological distancing), whether stimuli are presented pseudo-randomly or in blocks by experimental condition, and the content and intensity of the emotional stimuli. In particular, it will be important to explore whether these design variations differentially impact measures of reappraisal effectiveness depending on participants' age. A quantitative meta-analysis that systematically studies the impacts of these experimental design variations on findings would be a helpful next step.

Future research should also consider the potential effects of culture and socioeconomic status on the emergence and developmental course of cognitive reappraisal. For example, there is some evidence from studies with adults that the neural correlates of reappraisal differ between participants from European American, Chinese, and Mexican backgrounds (Qu and Telzer, 2017; Hampton et al., 2021). To examine whether cultural and ethnic differences influence the developmental course of cognitive reappraisal, we first need to increase the systematic reporting of the racial/ethnic and cultural identities of research participants. Likewise, a handful of studies have linked socioeconomic status to differences in the neural circuitry underlying emotion regulation in both adults and children (Hao and Farah, 2020), but little research has been conducted on socioeconomic differences in the developmental course of cognitive reappraisal.

The role of participant gender in reappraisal effectiveness is another avenue for further exploration. While research with adults has documented some neural differences (McRae et al., 2008), small sample sizes have limited the ability for studies of children to detect any psychophysiological gender effects (McRae et al., 2012a; Dougherty et al., 2015). Other studies have documented that adolescent girls use more cognitive reappraisal than boys (Zhao et al., 2014), and have suggested that the relative efficacy of emotion regulation strategies may vary for boys compared to girls (e.g., Zhang et al., 2020). Yet, whether the variations in reappraisal use and effectiveness by gender might also change with age (e.g. whether the gap in preference for reappraisal between men and women increases with age) is an area where additional research is needed.

The degree to which developmental changes in reappraisal use or effectiveness may vary depending on the discrete emotion being reappraised also deserves further investigation. While roughly one fifth of the studies included in this review assessed reappraisal targeting a discrete emotion such as sadness or anger, only four studies separately examined reappraisal targeting more than one discrete emotion in such a way that emotion-specific effects could be examined. Future research should more systematically explore how the emergence and developmental progression of cognitive reappraisal may differ depending on the discrete emotion being regulated, for example whether the emotion is aversive (e.g., anger, sadness) vs. appetitive (e.g., food craving).

Finally, future studies using a wider variety of methodologies—beyond self-report questionnaires and emotion regulation tasks—could provide new insights into the development of reappraisal in children and adolescents. For example, only a handful of studies used vignette-based (*n* = 4), interview (*n* = 3), experience sampling self-report (*n* =2), or daily diary (*n* =1) assessments of reappraisal. These methodologies deserve more attention. Experience sampling and daily diary methodologies offer a high level of ecological validity because they assess respondents' use of reappraisal on a typical day or moment in their everyday lives, and they are less subject to recall bias than are self-report questionnaires. However, these methodologies are also limited by respondents' level of introspective awareness and are most appropriate for use with adolescents. Vignette-based assessments may have greater ecological validity than many laboratory-based paradigms if the vignettes are designed to be relatable to the participant population. Vignette-based assessments are also more appropriate for younger children than are self-report questionnaires since they are less abstract, allowing for their use across a wider age range. Interview protocols as well as open-ended vignette-based assessments can yield rich data on participants' ability to generate reappraisals and the content of their reappraisals. Such methodologies would allow for the exploration of changes in the quantity, quality and content of reappraisals used across development.

## Implications

More work remains to be done to clarify the emergence and developmental course of cognitive reappraisal in children and adolescents, as findings vary depending on the methodology used. While experimental studies using observations and affective self-reports suggest that reappraisal is typically an effective strategy by middle childhood or earlier, results from studies using neural measures provide mixed evidence of reappraisal effectiveness prior to mid to late adolescence. Parents, teachers, and clinicians who wish to support children's development of this skill would benefit from a clear scientific consensus regarding the age at which reappraisal emerges as an effective emotion regulation strategy and how its use and effectiveness change from childhood through adolescence, as well as the individual factors influencing the emergence and developmental course of reappraisal. This knowledge could guide developmentally appropriate expectations for children's and adolescents' abilities to manage their emotions using reappraisal, with implications for social-emotional learning curricula and clinical practices. To achieve scientific consensus on this topic, we must first refine our methodology for measuring reappraisal to ensure our measures are objective, developmentally appropriate, and ecologically valid in early childhood through adolescence.

## Data Availability Statement

The original contributions presented in the study are included in the article/Supplementary Material, further inquiries can be directed to the corresponding author/s.

## Author Contributions

CW, JH, CB, AH, CC, and MB contributed to the conception and design of the systematic review. CW, JH, CB, AH, BG, and ZN wrote sections of the manuscript. CW directed the article identification, screening, and coding work. AH and BG screened and coded all articles included in the review. CW and AH conducted the quantitative analyses. CW, JH, and CB contributed to the narrative review of findings. All authors contributed to manuscript revisions and read and approved the submitted version.

## Funding

This work was supported by a grant from the Chan Zuckerberg Initiative DAF, an advised fund of the Silicon Valley Community Foundation.

## Conflict of Interest

The authors declare that the research was conducted in the absence of any commercial or financial relationships that could be construed as a potential conflict of interest.

## Publisher's Note

All claims expressed in this article are solely those of the authors and do not necessarily represent those of their affiliated organizations, or those of the publisher, the editors and the reviewers. Any product that may be evaluated in this article, or claim that may be made by its manufacturer, is not guaranteed or endorsed by the publisher.
