# Supplementary material for: The Development of Cognitive Reappraisal From Early Childhood Through Adolescence: A Systematic Review and Methodological Recommendations
Source: Front Psychol. 2022 Jun 22;13:875964. doi: 10.3389/fpsyg.2022.875964 (PMC9258621; doi:10.3389/fpsyg.2022.875964)
Supplement: Supplementary file 2 [file Table_2.DOCX]

**Search Terms and Filters**

**Stage 1 Article Search**

Listed below are the full search terms (query strings) and filters used within each database for the first stage of the article search, conducted on August 9^th^, 2018. Two separate keyword searches were completed within each database.

**Database: Scopus**

*Query Strings*

First Search

TITLE-ABS-KEY((child* OR adolesc* OR youth OR student OR preschool OR “pre-Kindergarten” OR kindergarten OR “elementary school” OR “middle school” OR “high school” OR “primary school” OR “secondary school” OR “early childhood” OR childcare) AND (((Emotion* OR affect* OR feeling OR mood OR anger OR disappointment OR frustration OR sadness OR fear OR anxiety OR boredom OR happiness) W/2 (regulat* OR downregulat* OR upregulat* OR manag* OR control OR suppress*)) OR “delay of gratification”) AND strateg* AND NOT (colleg* OR university OR undergrad*))

Second Search

TITLE-ABS-KEY((child* OR adolesc* OR youth OR student OR preschool OR “pre-Kindergarten” OR “elementary school” OR “middle school” OR “high school” OR “primary school” OR “secondary school” OR “early childhood” OR childcare) AND (“cognitive reappraisal” OR “positive self-talk” OR “situation modification” OR “support-seeking” OR “expressive suppression”) AND NOT (colleg* OR university OR undergrad*))

*Filters*

- Document type = Article or Article in Press
- Source Type = Journal
- Language = English
- Subject area = Psychology & Social Sciences

**Database: PsycINFO**

*Query Strings (searched within Abstracts & Titles)*

First Search

(((Emotion* OR affect* OR feeling OR mood OR anger OR disappointment OR frustration OR sadness OR fear OR anxiety OR boredom OR happiness) N2 (regulat* OR downregulat* OR upregulat* OR manag* OR control OR suppress*)) OR “delay of gratification”) AND strateg* NOT (colleg* OR university OR undergrad*)

Second Search

“cognitive reappraisal” OR “positive self-talk” OR “situation modification” OR “support-seeking” OR “expressive suppression” NOT (colleg* OR university OR undergrad*)

*Filters*

- Publication Type = Peer reviewed journals
- Age groups = preschool, school age, & adolescence
- Language = English
- Population group = human
- Document type = journal article
- Exclude dissertations

**Database: ERIC**

*Query Strings*

First Search

noft((((Emotion* OR affect* OR feeling OR mood OR anger OR disappointment OR frustration OR sadness OR fear OR anxiety OR boredom OR happiness) N/2 (regulat* OR downregulat* OR upregulat* OR manag* OR control OR suppress*)) OR “delay of gratification”) AND strateg* NOT (colleg* OR university OR undergrad*))

Second Search

noft(“cognitive reappraisal” OR “positive self-talk” OR “situation modification” OR “support-seeking” OR “expressive suppression” NOT (colleg* OR university OR undergrad*))

*Filters*

- Limit to peer-reviewed articles
- Document type = journal articles
- Language = English
- Education levels = Preschool, Kindergarten, Elementary School, Middle School, Junior High School, High School, Primary Education, & Secondary Education

**Stage 2 Article Search**

Using the EndNote library search feature, records retained from the first article search and screening stage were searched for inclusion of the following terms in the title or abstract: “reappraisal,” “cognitive restructuring,” “reframing,” “re-evaluating,” and “cognitive emotion regulation.” Only records including any of these terms in the title or abstract were retained.
